# FOXP1 transcriptionally activates NUSAP1 to induce M2 polarization of tumor-associated macrophages via glycolysis in hepatocellular carcinoma

**DOI:** 10.3389/fgene.2026.1817333

**Published:** 2026-08-24

**Authors:** Zhiwei Wang, Yuyi Mao, Jiangang Wang, Yafeng Yu, Jiawei Shao, Yingming Fei, Jinlong Zhu, Mingjiong Tong

**Affiliations:** 1 Hepatology Department II, Affiliated Hospital of Shaoxing University, Shaoxing, China; 2 Hepatology Department III, Affiliated Hospital of Shaoxing University, Shaoxing, China; 3 Department of Hepatobiliary and Pancreatic Surgery I, Affiliated Hospital of Shaoxing University, Shaoxing, China

**Keywords:** FOXP1, glycolysis, hepatocellular carcinoma, NUSAP1, tumor-associated macrophage M2 polarization

## Abstract

**Background:**

In hepatocellular carcinoma (HCC), the polarization of tumor-associated macrophages (TAMs) towards the M2 phenotype is a key factor driving the formation of an immunosuppressive microenvironment and malignant progression, a process frequently associated with glycolytic metabolic reprogramming. Nucleolar and spindle associated protein 1 (NUSAP1) is aberrantly overexpressed in various malignancies. However, whether it promotes HCC progression by driving TAM M2 polarization through regulating tumor cell glycolysis remains unclear.

**Methods:**

Bioinformatics analysis was used to examine NUSAP1 expression in HCC and its correlation with M2 TAM infiltration. qRT-PCR and Western blot were employed to validate NUSAP1 expression levels in HCC cell lines. Functional experiments, including flow cytometry, ELISA, colony formation, and Transwell assays, were conducted to investigate the regulatory effects of NUSAP1 on TAM M2 polarization and HCC cell malignant behaviors. GSEA and correlation analyses were performed to explore the association between NUSAP1 and the glycolysis pathway. The impact of NUSAP1 on glycolytic metabolic reprogramming in HCC cells was assessed via Seahorse energy metabolism analysis, ATP/lactate production assays, and detection of key glycolytic protein expression. Furthermore, bioinformatics analysis predicted the transcriptional regulation of NUSAP1 by forkhead box P1 (FOXP1), which was subsequently validated by ChIP and dual-luciferase reporter assays. Finally, *in vitro* rescue experiments and an allograft mouse tumor model were used to confirm that the FOXP1/NUSAP1 axis induces TAM M2 polarization via glycolysis and accelerates HCC progression.

**Results:**

NUSAP1 was significantly overexpressed in HCC, and its expression level positively correlated with M2 TAM infiltration. Functional experiments demonstrated that NUSAP1 facilitates HCC progression by driving glycolytic metabolic reprogramming in tumor cells and inducing polarization to the M2 phenotype. Mechanistically, FOXP1, as a transcription factor, binds to the NUSAP1 promoter region and activates its transcription. Both *in vitro* and *in vivo* experiments further confirmed that the FOXP1/NUSAP1 axis promotes TAM M2 polarization by activating glycolytic metabolism, thereby accelerating HCC progression.

**Conclusion:**

This study reveals a novel mechanism by which FOXP1 transcriptionally upregulates NUSAP1 expression, resulting in glycolytic metabolic reprogramming that induces TAM M2 polarization and promotes HCC progression. These findings identify a potential therapeutic avenue for reversing the immunosuppressive microenvironment in HCC.

## 1 Introduction

Liver cancer ranks as the sixth most common cancer worldwide and the third leading cause of cancer-related deaths, with approximately 865,000 new cases and 757,000 deaths annually (Bray et al., 2024). Hepatocellular carcinoma (HCC) constitutes over 80% of primary liver cancer cases (Ladd et al., 2024). The overall survival rate for HCC patients remains poor, with a median survival typically 6–10 months (Brar et al., 2020). Although emerging therapies such as targeted and immunotherapy show promise in metastatic HCC, the 5-year survival rate remains low (Yang et al., 2019; Yang et al., 2023). Therefore, exploring molecular mechanisms underlying HCC progression is critical for identifying novel diagnostic markers and therapeutic targets.

High HCC mortality is closely linked to high metastasis rates and therapy resistance, largely due to the immunosuppressive state of the tumor microenvironment (TME) (Chen Y. et al., 2025). Tumor-associated macrophages (TAMs), as key effector cells, hold a central role through functional polarization and diversity (Tian et al., 2019). In the TME, activated M1-type macrophages secrete inflammatory mediators like IL-1 and IL-6, promoting Th1-type immune responses and enhancing effector T cell function for anti-tumor immunity (Strizova et al., 2023). Conversely, M2 TAMs possess anti-inflammatory and immunoregulatory properties, evidenced by the production of IL-10 and the upregulation of markers including CD163, Arg1, and CD206. Through these mechanisms, these cells contribute to the establishment of an immunosuppressive environment and tumor progression (Yang et al., 2025). Notably, aerobic glycolysis, a core mechanism of metabolic reprogramming in tumor cells, can modulate M2 TAM polarization through its metabolite lactate (Feng et al., 2020). Under adequate oxygen, HCC cells preferentially undergo glycolysis, producing and releasing large amounts of lactate into the TME (Feng et al., 2020). This lactate can activate receptors on TAMs, initiating downstream signaling pathways that induce M2 polarization (Xu et al., 2024). Thus, targeting glycolysis-driven TAM M2 polarization may be a potential strategy to reverse TME immunosuppression and improve HCC treatment efficacy, the importance of identifying key molecular nodes that connect tumor glycolysis to immune remodeling.

Nucleolar and spindle associated protein 1 (NUSAP1), a critical mitotic regulator, shows aberrant expression associated with the development of various cancers (Xu et al., 2025). NUSAP1 is regulated by estrogen signaling in lung adenocarcinoma and promotes tumor growth and is identified as a potential therapeutic target for preventing non-alcoholic fatty liver disease progression to HCC(Zeng et al., 2022; Zhang et al., 2024). A pan-cancer analysis further indicates that NUSAP1 expression may serve as a prognostic and immunotherapeutic response biomarker in 26 cancers, including HCC(Zheng et al., 2023). Importantly, NUSAP1 promotes pancreatic ductal adenocarcinoma metastasis by activating LDHA-mediated glycolysis (Chen M. et al., 2023), indicating NUSAP1’s potential to influence tumor progression via glycolysis. However, whether and how NUSAP1 regulates glycolysis to affect TAM polarization in HCC requires further elucidation.

In conclusion, we elucidate that forkhead box P1 (FOXP1) functions as a transcription factor (TF) for NUSAP1 by integrating bioinformatics analyses with experimental validation both *in vitro* and *in vivo*. This upregulation drives glycolytic reprogramming and M2 polarization of TAMs, thereby accelerating HCC malignancy. These findings systematically underscore the critical importance of the FOXP1/NUSAP1/glycolysis axis, presenting a novel perspective for therapeutic targeting to enhance HCC immunotherapy.

## 2 Methods

### 2.1 Bioinformatics analysis

Initially, mRNA sequencing data for HCC were obtained from the TCGA database (Seyedian et al., 2019). The Wilcoxon rank-sum test was utilized to compare NUSAP1 mRNA expression between HCC and adjacent normal tissues. The correlation between NUSAP1 expression and M2 TAM infiltration levels in HCC was analyzed via the TIMER2 database (Veauthier and Hornecker, 2018). Based on the TCGA-LIHC dataset, samples were divided into NUSAP1 high/low-expression groups using the median expression value, followed by pathway enrichment analysis using GSEA software. Concurrently, we assessed relationships between NUSAP1 and key glycolytic genes (HK2, PDK1, LDHA, PFKM) and FOXP1 expression using Pearson correlation analysis. Potential upstream transcription factors targeting NUSAP1 were predicted using the hTFtarget database (Zhang et al., 2020). The JASPAR online platform (Caio et al., 2021) was applied to predict potential transcriptional regulation between NUSAP1 and FOXP1.

### 2.2 Cell culture and reagents

The human immortalized hepatocyte line THLE-3 was propagated in BEGM BulletKit medium (Lonza, Switzerland) with 1% P/S (Solarbio, China) and 10% FBS (Gibco, USA). In contrast, human HCC cell lines (HCCLM3, Huh-7), mouse HCC cell line (Hepa 1-6), and human embryonic kidney 293T cells were cultured in DMEM medium (Gibco, USA) containing 1% P/S and 10% FBS. For the SNU-182 cell line, RPMI 1640 medium (Gibco, USA) with 1% P/S and 10% FBS was used. Human acute monocytic leukemia THP-1 cells were grown in THP-1-specific medium (RPMI 1640 containing 1% P/S, 10% FBS, and 0.05 mM β-mercaptoethanol) (BNCC, China).

THP-1 cells were differentiated into M0 TAMs using 200 ng/mL phorbol myristate acetate (PMA, MCE, USA). M0 TAMs were then cultured with conditioned medium (CM) from centrifuged HCC cell supernatants. All cells were maintained at 37 °C with 5% CO_2_ (Thermo Fisher, USA). The cells used are listed in Table 1.

**TABLE 1:** Cell information.

Cell name	Catalog number	Supplier
Human immortalized hepatocyte THLE-3	GNHu40	Cell bank of type culture collection of Chinese academy of sciences (CBTCCCAS), China
Human HCC cell line HCCLM3	SCSP-5093	CBTCCCAS, China
Human HCC cell line SNU-182	SCSP-5047	CBTCCCAS, China
Human HCC cell line Huh-7	TCHu182	CBTCCCAS, China
Human acute monocytic leukemia THP-1	BNCC358410	BNCC, China
Mouse HCC cell line hepa 1-6	SCSP-512	CBTCCCAS, China
Human embryonic kidney 293T cells	SCSP-502	CBTCCCAS, China

### 2.3 RNA interference, plasmid construction, and viral infection

Small interfering RNAs (siRNAs) targeting NUSAP1 and FOXP1 (si-NUSAP1, si-NUSAP1#2, si-FOXP1), short hairpin RNAs (shRNAs) (sh-NUSAP1), negative controls (si-NC, sh-NC), overexpression plasmids for NUSAP1 and FOXP1 (oe-NUSAP1, oe-FOXP1), and corresponding empty vector controls (oe-NC) were synthesized by GenePharma (China). Cell transfection was accomplished using Lipofectamine 2000 (Invitrogen, USA). The efficiency of gene knockdown or overexpression was quantified via qRT-PCR 48–72 h following cell transfection.

Lentivirus packaging involved co-transfecting 293T cells with the lentiviral backbone pLVX-Puro (Youbio, China), psPAX2 packaging plasmid (Sangon Biotech, China), and pCMV-VSV-G envelope plasmid (Beyotime, China) using Lipofectamine 3000 (Invitrogen, USA). Virus-containing supernatant was collected 48 h post-transfection, filtered through a 0.45 μm membrane, and applied to infect mouse HCC Hepa 1-6 cells to establish stable cell lines.

### 2.4 Western blot (WB)

Cell lysates were prepared using RIPA buffer (Yeasen, China) containing protease and phosphatase inhibitors, and protein concentration was determined with a BCA protein assay kit (Thermo Fisher, USA). Following separation by SDS-PAGE, proteins were blotted onto PVDF membranes (Millipore, USA). Subsequent blocking in 5% skim milk (1 h) at room temperature (RT) preceded overnight incubation with primary antibodies (Table 2) at 4 °C. After rinsing, samples were exposed to HRP-conjugated secondary antibodies (Table 2) for 1 h at RT. Signals were detected using ECL substrate (Thermo Fisher, USA).

**TABLE 2:** Antibody information.

Antibody	Catalog number	Supplier
Rabbit anti-NUSAP1 antibody	PA5-106697	Invitrogen, USA
Rabbit anti-HK2 antibody	ab209847	Abcam, UK
Rabbit anti-PDK1 antibody	ab202468	Abcam, UK
Rabbit anti-LDHA antibody	ab300638	Abcam, UK
Rabbit anti-PFKM antibody	ab237545	Abcam, UK
Rabbit anti-FOXP1 antibody	ab314488	Abcam, UK
Rabbit anti-β-actin antibody	ab213262	Abcam, UK
Goat anti-rabbit IgG H&L (HRP)	ab205718	Abcam, UK

### 2.5 qRT-PCR

Total RNA was extracted using the ESscience RNA Rapid Purification Kit (Yishan Biotechnology, China). RNA was subjected to reverse transcription into cDNA by implementing the HiScript II Q RT SuperMix for qPCR Kit (Vazyme, China). qPCR was performed using SYBR Green Master Mix (Vazyme, China) and specific primers (Table 3) on an ABI 7500 Real-Time PCR System (Thermo Fisher, USA). Relative mRNA expression was quantified by the 2^−ΔΔCT^ method, with β-actin as an internal control. Primers were synthesized by Tsingke Biotechnology (China).

**TABLE 3:** qRT-PCR Primer Sequences.

Primer	Forward (5’-3’)	Reverse (5’-3’)
NUSAP1	TGT​GGA​CCC​TGA​CTC​ACA​GA	TCT​CAG​CTT​CTT​GGT​GCT​CG
FOXP1	TTC​AAG​CCC​TTG​GGA​GTT​GG	ATG​GGG​AAG​GGT​TAG​GCT​GA
β-actin	TTC​ACA​ATG​TGG​CCG​AGG​A	AGT​GGG​GTG​GCT​TTT​AGG​ATG

### 2.6 Flow cytometry (FCM)

For TAM phenotype detection, THP-1-derived TAMs were stained with PE-conjugated mouse anti-human CD163 (A15792, Thermo Fisher, USA) and PE-Cy7-conjugated mouse anti-human CD206 (25-2069–42, Thermo Fisher, USA). Mouse tumor tissue single-cell suspensions were stained with FITC-conjugated rabbit anti-mouse CD206 (FITC-98031, Proteintech, China) and APC-conjugated rat anti-mouse CD163 (17-1631–82, Invitrogen, USA). Samples were resuspended in ice-cold PBS with 10% FBS, incubated with antibodies 4 °C for 30 min, protected from light. After discarding the supernatant via centrifugation at 400 *g* to eliminate free antibodies, the samples were analyzed using a flow cytometer (Agilent, USA).

For apoptosis analysis, HCC cells (5 × 10^5^ per well in 6-well plates) were collected after 24 h and processed using an Annexin V-FITC/PI Apoptosis Detection Kit (BD Biosciences, USA). After a 15-min incubation at RT, avoiding light exposure, samples were analyzed by a flow cytometer (Agilent, USA).

### 2.7 ELISA

ELISA kits were applied to measure levels of M2 TAM-related cytokines Arg1 and IL-10 in HCC cell culture supernatants and mouse serum. Human Arg1 ELISA Kit (FineTest, China), human IL-10 ELISA Kit (FineTest, China), mouse Arg1 ELISA Kit (Abcam, UK), and mouse IL-10 ELISA Kit (FineTest, China) were used. Optical density (OD) at 450 nm was measured using a microplate reader (BioTek, USA), and cytokine concentrations were calculated based on standard curves.

### 2.8 Colony formation assay for cell proliferation

HCC cells (800 cells per dish) were seeded in 6 cm dishes with 10% FBS medium and cultured for 10 days, with medium changed every 3 days. Following fixation with 4% PFA (Beyotime, China) for 15 min, colonies were stained with 0.1% crystal violet (Beyotime, China) for 30 min. Finally, samples were imaged under an optical microscope (Olympus, Japan) and counted using ImageJ software (NIH, USA).

### 2.9 Transwell assay for cell migration and invasion

Cell migration and invasion were assessed using 24-well Transwell systems with 8.0 μm pores (Corning, USA). HCC cells (2 × 10^4^ cells/mL, 100 μL) were inoculated into the upper chamber. After adhesion, the upper chamber medium was replaced with serum-free conditioned medium from respective experimental groups, while the lower counterpart contained complete medium with 10% FBS as a chemoattractant. After incubation for 24 h (migration) or 36 h (invasion) at 37 °C, cells were subjected to fixation with 4% PFA, staining with 0.1% crystal violet, and non-migrated cells on the upper surface were removed with a cotton swab. Migrated/invaded cells were imaged and counted under an optical microscope (Olympus, Japan). For invasion assays, the upper chamber was pre-coated with Matrigel matrix (Corning, USA) and solidified at 37 °C for 1 h before the experiment.

### 2.10 Extracellular acidification rate (ECAR) measurement

The glycolytic capacity of HCC cells was assessed using the Seahorse XFe 96 Analyzer (Agilent, USA) and the XF Glycolysis Stress Test Kit (Agilent, USA). Initially, cells (1.5 × 10^4^ per well) were seeded in Seahorse microplates. At approximately 90% confluence, culture medium was replaced with XF assay medium (Agilent, USA) and equilibrated for 1 h at 37 °C in a non-CO_2_ incubator. After baseline measurement, 10 mM glucose, 1 μM oligomycin, and 50 mM 2-DG were sequentially injected. ECAR was recorded in mpH/min.

### 2.11 ATP measurement

Intracellular ATP levels were measured using an ATP Assay Kit (Solarbio, China). Briefly, HCC cells (5 × 10^6^ cells/well in 12-well plates) were lysed for 24 h. After washing with PBS and removing the medium, cells were lysed with lysis buffer (200 μL). Cell lysates were centrifuged at 10,000×g for 10 min at 4 °C. The collected supernatant was mixed with 500 μL chloroform, followed by a second centrifugation step at 10,000×g for 3 min at 4 °C. The supernatant was combined with ATP detection working solution, and OD at 340 nm was measured using a microplate reader (BioTek, USA) to calculate ATP concentration.

### 2.12 Lactate measurement

HCC cells (2 × 10^5^ cells/well in 6-well plates) were cultured in fresh medium with 5 mM glucose for 15 h. Culture supernatants were collected, and lactate concentration was determined using an L-Lactate Assay Kit (Solarbio, China). Reagents were mixed and incubated at 37 °C for 10 min, protected from light. After adding reagent 5, 200 μL of reaction mixture was transferred to a 96-well plate, and OD at 570 nm was measured. For mouse tumor tissue lactate content, tumor tissues were homogenized on ice, centrifuged at 12,000 g for 10 min at RT, and the supernatant was assayed similarly.

### 2.13 ChIP

HCC cells in dishes were cross-linked with 1% formaldehyde (Sigma-Aldrich, USA) for 10 min at RT, quenched with 0.125 M glycine. After treatment in lysis buffer (50 mM Tris-HCl, pH 8.0, 0.5 mM EDTA, 1% SDS, with protease inhibitors), cells were sonicated to shear the chromatin into 300–500 bp fragments. Reagents were from Sigma-Aldrich (Merck, Germany). Immunoprecipitation was performed using FOXP1 antibody (PA5-52006, Thermo Fisher, USA), with IgG (MA5-56524, Thermo Fisher, USA) as a negative control. Precipitated DNA was purified and analyzed by qPCR with specific primers to detect enrichment of NUSAP1 promoter fragments.

### 2.14 Dual-luciferase reporter assay

Genomic fragments containing the NUSAP1 promoter region were cloned into the pGL3-basic vector (YouBio, China) to construct wild-type and mutant reporter plasmids. Reporter plasmids were co-transfected with the pRL-TK Renilla luciferase internal control plasmid (YouBio, China) into cells along with si-NC (negative control) or si-FOXP1 (FOXP1 knockdown) constructs. After 48 h, luciferase activity was measured using a Dual-Luciferase Reporter Assay Kit (Beyotime, China). Firefly luciferase activity was normalized to Renilla luciferase activity.

### 2.15 Allograft mouse model

Six-to eight-week-old female C57BL/6 mice (Beijing Vital River Laboratory Animal Technology Co., Ltd., China) were used. All animal procedures were approved by the Animal Ethics Committee of Zhejiang Luoxi Medical Technology Co., Ltd., Hangzhou, China, APPROVAL NO. LX4825040702. Mice were housed under specific pathogen-free conditions with free access to food and water. Stable mouse HCC Hepa1-6 cell lines transfected with oe-NC + sh-NC, oe-FOXP1+sh-NC, oe-NC + sh-NUSAP1, or oe-FOXP1+sh-NUSAP1 were prepared as single-cell suspensions and subcutaneously inoculated into the right dorsal flank of mice (5 × 10^6^ cells per mouse, n = 6 per group). Tumor dimensions (short and long diameters) were measured every 5 days starting from day 5 until day 25. Mice were then euthanized, tumors were dissected and weighed, and tumor tissue and serum samples were collected for subsequent analysis.

### 2.16 IHC analysis

Paraffin-embedded tumor tissue sections were baked at 60 °C for 3 h, deparaffinized in xylene (Merck, Germany), rehydrated through graded ethanol (100%–70%, Sinopharm, China), and antigen retrieval was performed using citrate buffer (Solarbio, China). Endogenous peroxidase was blocked with 3% H_2_O_2_ (Sinopharm, China) for 30 min. After PBS washes, sections were blocked with 5% goat serum (Solarbio, China) and incubated with primary antibodies (Table 4) against FOXP1, NUSAP1, Ki67, and HK2 at 4 °C overnight. After incubation with secondary antibodies (Table 4) at RT for 30 min, sections were developed with DAB (Beyotime, China), counterstained with hematoxylin (Solarbio, China), and imaged using an inverted microscope (Nikon, Japan).

**TABLE 4:** Antibody information for IHC.

Antibody	Catalog number	Supplier
Rabbit anti-NUSAP1 antibody	PA5-106697	Invitrogen, USA
Rabbit anti-FOXP1 antibody	ab314488	Abcam, UK
Rabbit anti-HK2 antibody	ab209847	Abcam, UK
Rabbit anti-Ki67 antibody	ab16667	Abcam, UK
Goat anti-rabbit IgG H&L (HRP)	ab205718	Abcam, UK

### 2.17 Terminal deoxynucleotidyl transferase (TdT) dUTP nick-end labeling (TUNEL) staining

Apoptosis in tumor tissues was assessed by TUNEL assay. Deparaffinized and rehydrated tissue sections were processed using a TUNEL (Fluorescence) Detection Kit (Servicebio, China). After incubation with TdT enzyme reaction mixture at 37 °C for 60 min, protected from light, sections were counterstained with DAPI and mounted with anti-fade mounting medium. Ultimately, samples were observed under a fluorescence microscope (Olympus, Japan).

### 2.18 Statistical analysis

Data were analyzed and graphed using GraphPad Prism 8.0 (GraphPad Software Inc., USA). Measurement data are presented as mean ± SD (n ≥ 3). Intergroup comparisons employed Student’s t-test (two groups) and one-way ANOVA (≥3 groups), with *P*-values <0.05 denoting significance.

## 3 Results

### 3.1 NUSAP1 is overexpressed in HCC and induces TAM M2 polarization

**FIGURE 1:**
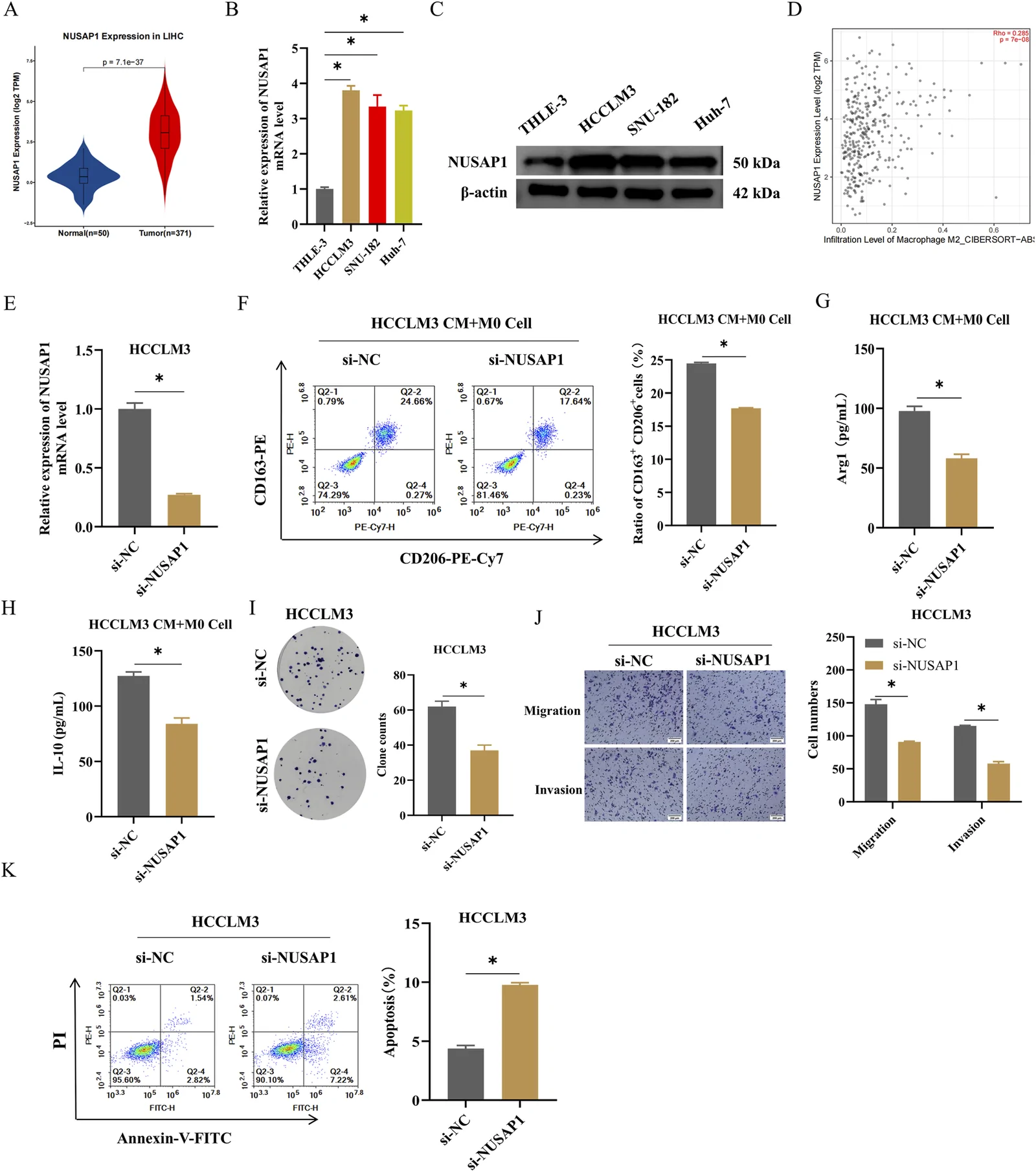
NUSAP1 Is Overexpressed in HCC and Induces TAM M2 Polarization. **(A)** NUSAP1 mRNA expression in human HCC and adjacent normal tissues from the TCGA-LIHC dataset. **(B)** qRT-PCR detection of NUSAP1 mRNA expression in THLE-3 and human HCC cell lines (HCCLM3, SNU-182, Huh-7). **(C)** Western blot detection of NUSAP1 protein expression in THLE-3 and human HCC cell lines. **(D)** TIMER2 database analysis showing correlation between NUSAP1 expression and M2 TAM infiltration levels. Cell groups: si-NC, si-NUSAP1. **(E)** qRT-PCR verification of transfection efficiency. CM from these cells was used to culture M0 TAMs. **(F)** Flow cytometry analysis of CD206^+^/CD163^+^ positive cell proportion. **(G,H)** ELISA detection of Arg1 **(G)** and IL-10 **(H)** release levels in cell supernatant. THP-1 cell supernatant was collected to culture HCC cells. **(I)** Colony formation assay for tumor cell proliferation. **(J)** Transwell assay for cell migration and invasion. **(K)** Flow cytometry for apoptosis analysis. *, *P* < 0.05; ns, not significant.

To investigate the role of NUSAP1 in the HCC immune microenvironment, we first analyzed the TCGA-LIHC dataset. The results showed significantly higher NUSAP1 mRNA expression in HCC tissues compared to adjacent normal tissues (Figure 1A). Subsequently, the human immortalized hepatocyte line THLE-3 and multiple HCC cell lines (HCCLM3, SNU-182, Huh-7) were cultured. qRT-PCR and WB confirmed significantly upregulated NUSAP1 mRNA and protein levels in these HCC cell lines relative to THLE-3 cells (Figures 1B,C). To further explore NUSAP1’s immunomodulatory function, immune infiltration analysis using the TIMER2 database showed a significant positive correlation between NUSAP1 expression levels and M2 TAM infiltration in HCC (Figure 1D), suggesting NUSAP1 involvement in modulating TAM M2 polarization. Based on these findings, HCCLM3 cells, which exhibited relatively higher NUSAP1 expressions, were selected for subsequent NUSAP1 knockdown functional experiments. A transient NUSAP1 knockdown model (si-NUSAP1) was successfully established, with knockdown efficiency confirmed by qRT-PCR (Figure 1E). To rule out potential off-target effects of siRNA, an additional independent siRNA (si-NUSAP1#2) targeting a distinct region of the NUSAP1 coding sequence was designed, which achieved a comparable knockdown efficiency (Supplementary Figure S1A). Conditioned medium (CM) from si-NC and si-NUSAP1 HCC cells was collected to treat PMA-induced M0 THP-1 macrophages. FCM revealed a notably reduced proportion of CD206^+^/CD163^+^ cells (M2 TAM markers) in the si-NUSAP1 CM-treated group compared to the si-NC CM-treated group (Figure 1F), an effect that was recapitulated when CM from cells transfected with a second, independent siRNA (si-NUSAP1#2) was used (Supplementary Figure S1B). Concurrently, ELISA showed decreased secretion levels of M2 TAM-related cytokines Arg1 and IL-10 in the supernatant of the si-NUSAP1 group (Figures 1G,H). These results indicate that NUSAP1 knockdown in tumor cells effectively inhibits TAM M2 polarization. To investigate the impact of TAM M2 polarization on HCC cell malignant behavior, culture supernatants from the differently treated M0 TAMs (induced by si-NC or si-NUSAP1 HCC CM) were collected and used to culture HCCLM3 cells. Compared to the si-NC group, cells treated with supernatant from the si-NUSAP1 group exhibited pronouncedly reduced colony formation capacity (Figure 1I), inhibited migration and invasion (Figure 1J), and increased apoptosis rate (Figure 1K). Collectively, NUSAP1 knockdown suppresses HCC cell malignant behaviors by inhibiting TAM M2 polarization.

### 3.2 NUSAP1 promotes glycolytic reprogramming in HCC cells to induce TAM M2 polarization

**FIGURE 2:**
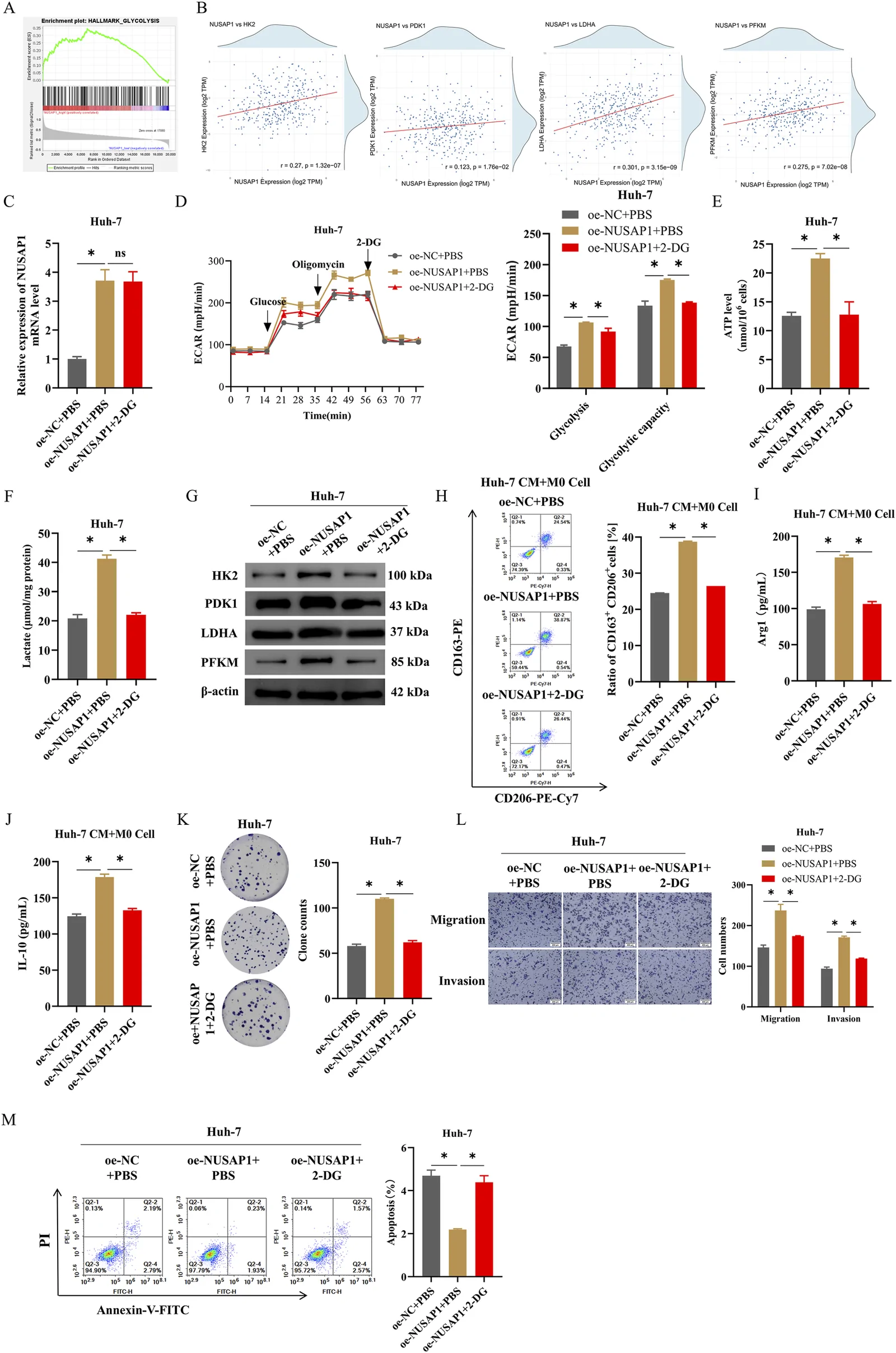
NUSAP1 Promotes Glycolytic Reprogramming in HCC Cells to Induce TAM M2 Polarization. **(A)** GSEA analysis of enriched pathways based on NUSAP1 differential expression. **(B)** TCGA database analysis of correlations between NUSAP1 and glycolytic genes (HK2, PDK1, LDHA, PFKM). Cell groups: oe-NC + PBS, oe-NUSAP1+PBS, oe-NUSAP1+2-DG. **(C)** qRT-PCR verification of transfection efficiency. **(D)** ECAR measurement using Seahorse XFe 96 Analyzer. **(E)** ATP production assay. **(F)** Lactate production assay. **(G)** Western blot detection of key glycolytic proteins HK2, PDK1, LDHA, and PFKM. Conditioned medium (CM) from these cells was used to culture M0 TAMs. **(H)** Flow cytometry analysis of CD206^+^/CD163^+^ positive cell proportion. **(I,J)** ELISA detection of M2 TAM-related cytokine Arg1 **(I)** and IL-10 **(J)** release levels. After 48 h, THP-1 cell supernatant was collected to culture HCC cells. **(K)** Colony formation assay for tumor cell proliferation. **(L)** Transwell assay for migration and invasion. **(M)** Flow cytometry for apoptosis analysis. *, *P* < 0.05; ns, not significant.

NUSAP1 has been reported to promote pancreatic cancer metastasis by activating LDHA-mediated glycolysis (Chen M. et al., 2023). Furthermore, the glycolytic pathway can induce TAM M2 polarization in HCC(Ji et al., 2023). Therefore, we hypothesized that in HCC, NUSAP1 might drive TAM M2 polarization by promoting glycolytic reprogramming, thereby influencing the tumor immune microenvironment (TIME). To test this, GSEA analysis was performed on TCGA HCC samples stratified into high and low NUSAP1 expression groups. Results showed substantial enrichment of the glycolysis signaling pathway (Figure 2A). Further analysis revealed significant positive correlations between NUSAP1 expression and several key glycolytic genes (HK2, PDK1, LDHA, PFKM) (Figure 2B).

To investigate the regulatory role of NUSAP1 in HCC glycolysis at the cellular level, NUSAP1-overexpressing (oe-NUSAP1) and negative-control (oe-NC) models were established in Huh-7 cells, and an additional group treated with the glycolysis inhibitor 2-deoxy-D-glucose (2-DG) was included. qRT-PCR confirmed efficient NUSAP1 overexpression (Figure 2C). Seahorse XF analysis showed remarkably increased ECAR in oe-NUSAP1 cells relative to controls, which was reversed by 2-DG treatment (Figure 2D). Consistently, ATP and lactate levels were markedly elevated in oe-NUSAP1 cells (Figures 2E,F), along with upregulated protein expression of key glycolytic enzymes HK2, PDK1, LDHA, and PFKM (Figure 2G). All these increases were reversed by 2-DG. These findings suggest NUSAP1 accelerates the glycolytic process in HCC cells.

Furthermore, CM from the above HCC cell groups was used to culture M0 TAMs to investigate whether NUSAP1-induced glycolysis participates in TAM M2 polarization. FCM showed a significantly increased proportion of CD206^+^/CD163^+^ cells in the oe-NUSAP1 group compared to control, an effect inhibited by 2-DG treatment (Figure 2H). ELISA revealed elevated Arg1 and IL-10 levels in the supernatant of the oe-NUSAP1 group, which were reversed by 2-DG (Figures 2I,J). Finally, supernatants from these differently conditioned TAMs were used to culture HCC cells to assess the impact of NUSAP1-mediated TAM M2 polarization on HCC malignant phenotypes. Colony formation, Transwell, and FCM assays collectively demonstrated that supernatants from oe-NUSAP1-group TAMs significantly promoted HCC cell proliferation (Figure 2K), migration and invasion (Figure 2L), and inhibited apoptosis (Figure 2M), effects reversed by 2-DG treatment. In summary, NUSAP1 activates the glycolytic pathway, promoting TAM M2 polarization, which in turn enhances HCC cell proliferation, migration, and invasion while inhibiting apoptosis.

### 3.3 FOXP1 upregulates its expression in HCC by transcriptionally activating NUSAP1

**FIGURE 3:**
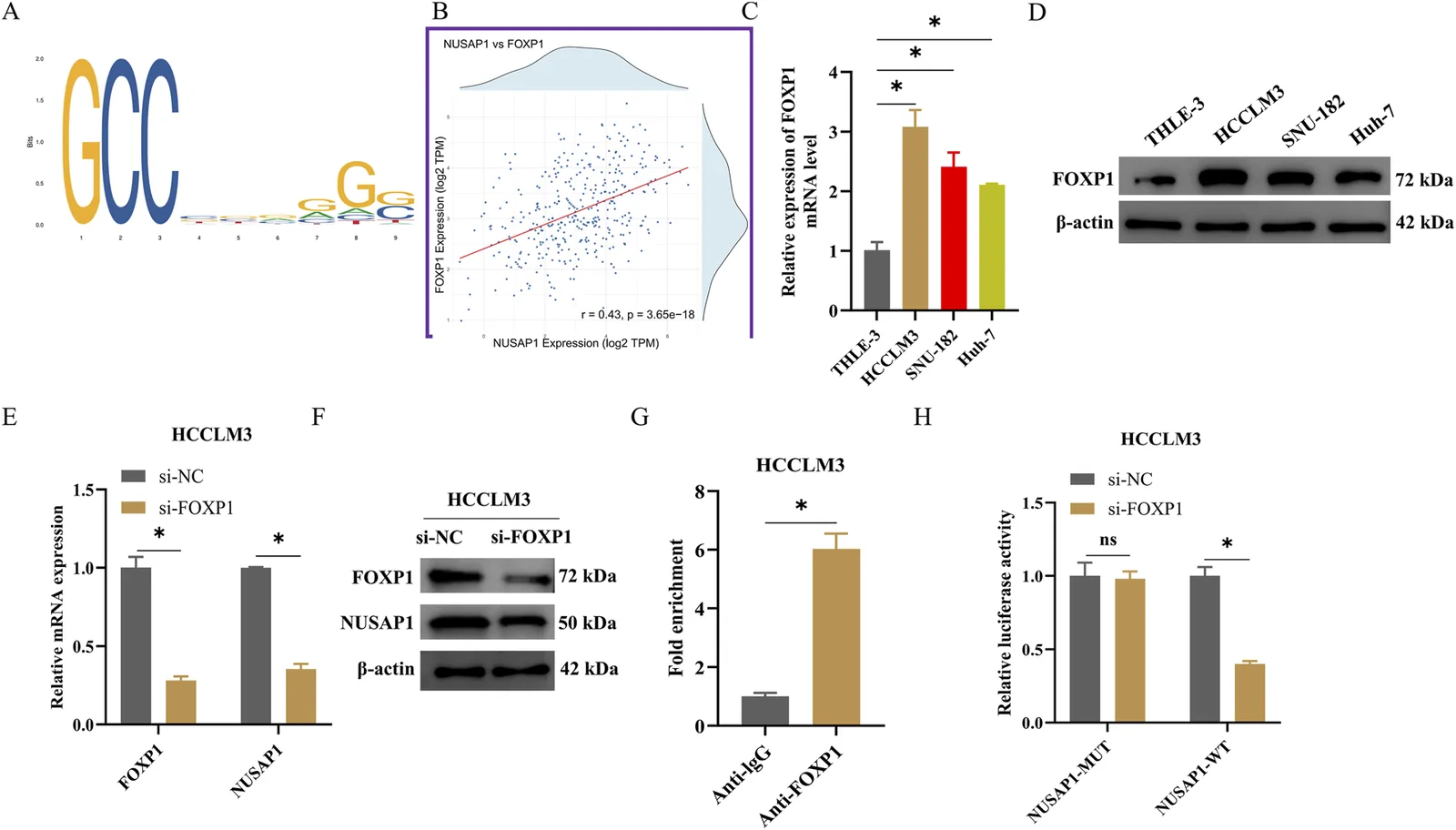
FOXP1 Upregulates Its Expression in HCC by Transcriptionally Activating NUSAP1. **(A)** JASPAR website analysis of transcriptional regulation between NUSAP1 and FOXP1. **(B)** TCGA database analysis of the correlation between FOXP1 and NUSAP1 expression. **(C)** qRT-PCR detection of FOXP1 mRNA expression in human THLE-3 and HCC cell lines (HCCLM3, SNU-182, Huh-7). **(D)** Western blot detection of FOXP1 protein expression in THLE-3 and HCC cell lines. Cell groups: si-NC, si-FOXP1. **(E)** qRT-PCR detection of FOXP1 and NUSAP1 mRNA expression. **(F)** Western blot detection of FOXP1 and NUSAP1 protein expression. **(G)** ChIP validation of FOXP1 binding to the NUSAP1 promoter. **(H)** Dual-luciferase assay validating FOXP1 transcriptional regulation of NUSAP1. *, *P* < 0.05; ns, not significant.

To explore upstream mechanisms regulating NUSAP1 overexpression, potential upstream TFs were analyzed via the hTFtarget database, identifying FOXP1. JASPAR prediction indicated a binding site for FOXP1 within the NUSAP1 promoter region (Figure 3A). Multiple studies have demonstrated that FOXP1 is significantly upregulated in HCC tissues compared with adjacent non-tumor tissues and acts as an oncogenic driver as well as an independent prognostic factor (Zhang et al., 2012; Ouyang et al., 2022). Moreover, existing research shows FOXP1, as a key TF, holds core regulatory roles in tumor cell metabolic reprogramming and TAM polarization (Wang et al., 2016; Chen X. et al., 2023; Konig et al., 2025). Therefore, we hypothesized FOXP1 might act as an upstream transcriptional regulator of NUSAP1 in HCC, influencing TAM M2 polarization. Further analysis of the TCGA database revealed a marked positive correlation between FOXP1 and NUSAP1 mRNA expression in HCC tissues (Figure 3B). qRT-PCR and WB analysis showed markedly upregulated FOXP1 mRNA and protein expression in HCCLM3, SNU-182, and Huh-7 cells compared to THLE-3 cells (Figures 3C,D), further confirming their positive correlation. A FOXP1 knockdown model (si-FOXP1) and negative control (si-NC) were constructed in HCCLM3 cells. As shown by qRT-PCR and WB, mRNA and protein levels of both FOXP1 and NUSAP1 were notably decreased in the si-FOXP1 group compared to si-NC (Figures 3E,F), indicating FOXP1 positively regulates NUSAP1 expression. Finally, ChIP and dual-luciferase assays confirmed their direct transcriptional binding relationship (Figures 3G,H). These results demonstrate that FOXP1 transcriptionally activates NUSAP1, thereby upregulating its expression in HCC.

### 3.4 FOXP1 activates NUSAP1 to induce M2 TAM polarization via glycolysis in HCC

**FIGURE 4:**
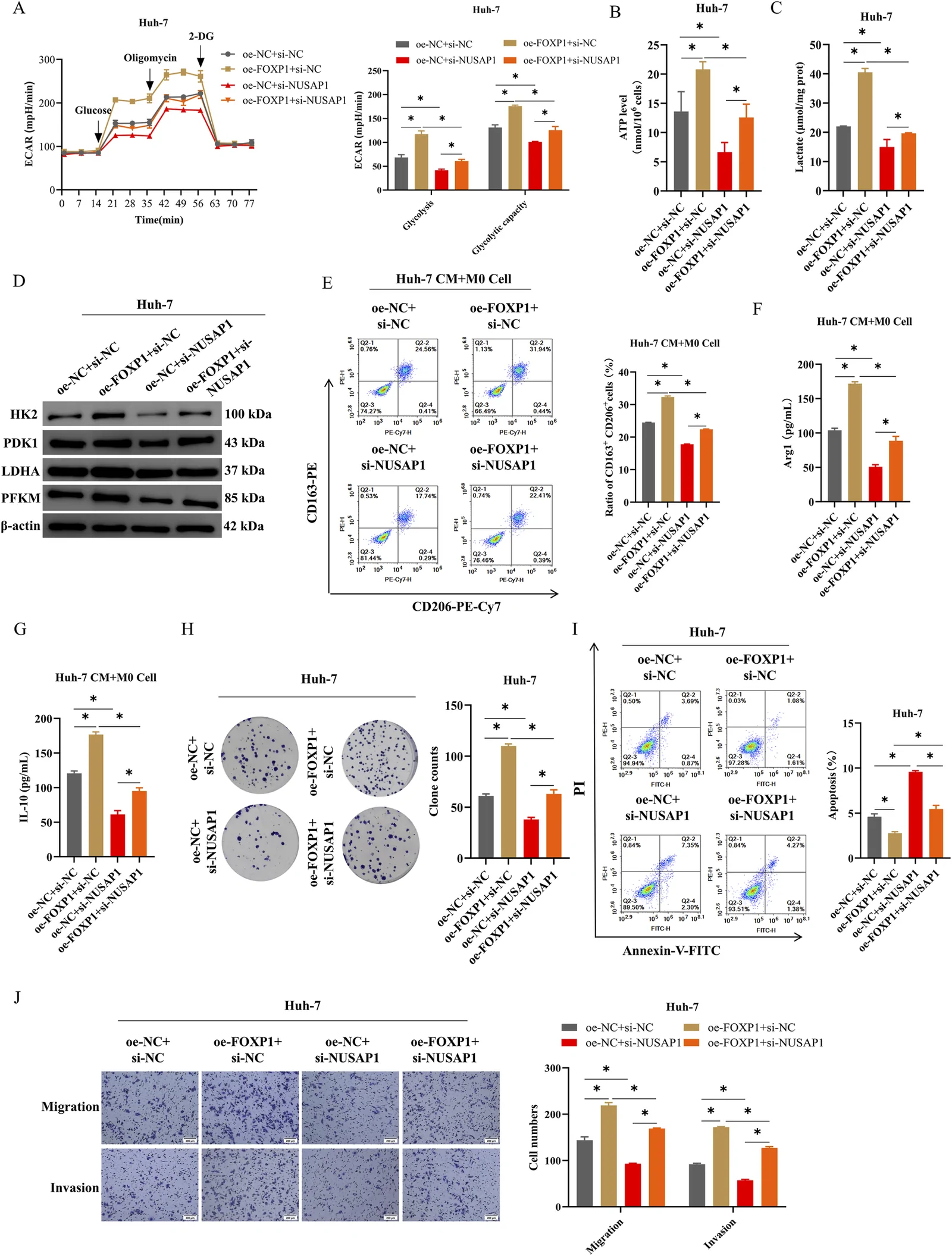
FOXP1 Activates NUSAP1 to Induce M2 TAM Polarization via Glycolysis in HCC. Cell groups: oe-NC + si-NC, oe-FOXP1+si-NC, oe-NC + si-NUSAP1, oe-FOXP1+si-NUSAP1. **(A)** ECAR measurement using Seahorse XFe 96 Analyzer. **(B)** ATP production assay. **(C)** Lactate production assay. **(D)** Western blot detection of HK2, PDK1, LDHA, and PFKM protein expression. CM from these cells was used to culture M0 TAMs. **(E)** Flow cytometry analysis of CD206^+^/CD163^+^ positive cell proportion. **(F,G)** ELISA detection of M2 TAM cytokines Arg1 **(F)** and IL-10 **(G)** release levels. After 48 h, THP-1 cell supernatant was collected to culture HCC cells. **(H)** Colony formation assay for tumor cell proliferation. **(I)** Flow cytometry for apoptosis analysis. **(J)** Transwell assay for migration and invasion. *, *P* < 0.05; ns, not significant.

To determine whether FOXP1 influences the HCC TME through NUSAP1-mediated glycolysis, four groups of Huh-7 cell models were constructed for validation. Metabolic function assays demonstrated substantially increased ECAR in oe-FOXP1+si-NC cells compared to oe-NC + si-NC controls, an effect markedly reversed by concurrent NUSAP1 knockdown (oe-FOXP1+si-NUSAP1) (Figure 4A). Correspondingly, ATP and lactate production were significantly elevated in the oe-FOXP1+si-NC group compared to oe-NC + si-NC, and this increase was suppressed by NUSAP1 knockdown (Figures 4B,C). WB further indicated that FOXP1 overexpression upregulated HK2, PDK1, LDHA, and PFKM protein expression, which was effectively blocked by NUSAP1 knockdown (Figure 4D). Subsequently, FCM revealed a significantly higher proportion of CD206^+^/CD163^+^ cells in TAMs cultured with CM from the oe-FOXP1+si-NC group relative to the oe-NC + si-NC group. This promoting effect was significantly inhibited in the oe-FOXP1+si-NUSAP1 group (Figure 4E). Additionally, ELISA showed increased secretion levels of Arg1 and IL-10 in the supernatant of the oe-FOXP1+si-NC group, which were markedly suppressed by NUSAP1 knockdown (Figures 4F,G).

To further investigate the impact of this TAM polarization on tumor malignant behavior, supernatants from differently conditioned TAMs were used to culture HCC cells. Colony formation, Transwell, and FCM assays collectively demonstrated that supernatants from oe-FOXP1+si-NC group TAMs significantly promoted HCC cell proliferation (Figure 4H), inhibited apoptosis (Figure 4I), and enhanced migration and invasion (Figure 4J) relative to the oe-NC + si-NC group. These promoting effects on HCC cell malignant phenotypes were effectively reversed by NUSAP1 knockdown in the context of FOXP1 overexpression (oe-FOXP1+si-NUSAP1) (Figures 4H–J). Collectively, FOXP1 upregulates NUSAP1 expression to activate glycolysis, thereby inducing TAM M2 polarization and ultimately promoting HCC progression.

### 3.5 *In vivo* validation that FOXP1 transcriptionally upregulates NUSAP1 to promote HCC progression

**FIGURE 5:**
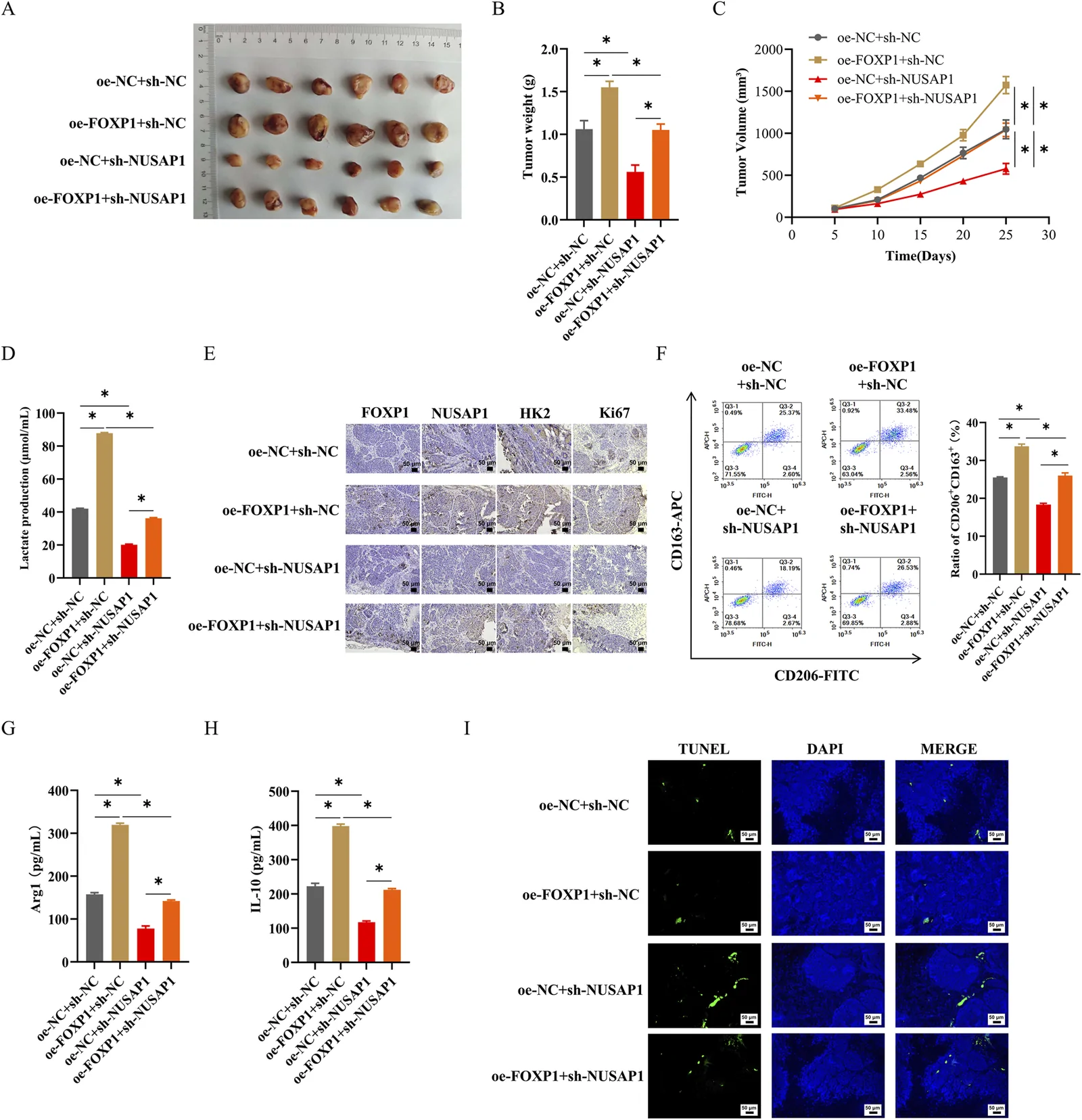
*In Vivo* Validation that FOXP1 Transcriptionally Upregulates NUSAP1 to Promote HCC Progression. Animal groups: oe-NC + sh-NC, oe-FOXP1+sh-NC, oe-NC + sh-NUSAP1, oe-FOXP1+sh-NUSAP1. **(A)** Representative images of allograft mouse tumors. **(B)** Tumor weights for each group. **(C)** Tumor volume changes over time for each group. **(D)** Lactate levels in tumor tissues. **(E)** IHC detection of FOXP1, NUSAP1, HK2, and Ki67 protein expression in tumor tissues. **(F)** Flow cytometry analysis of CD206^+^/CD163^+^ positive cell proportion. **(G,H)** ELISA detection of M2 TAM-related cytokines Arg1 **(G)** and IL-10 **(H)** release levels. **(I)** TUNEL staining for apoptosis in tumor tissues. *, *P* < 0.05; ns, not significant.

Finally, to validate the role of the FOXP1/NUSAP1 axis in HCC progression *in vivo*, a subcutaneous allograft mouse model was established using stably transfected mouse HCC Hepa 1-6 cells in C57BL/6 mice. Tumor growth monitoring revealed significantly accelerated tumor growth in the oe-FOXP1+sh-NC group compared to the oe-NC + sh-NC control group, while tumor growth was markedly inhibited in the oe-NC + sh-NUSAP1 group. Notably, NUSAP1 knockdown in the context of FOXP1 overexpression (oe-FOXP1+sh-NUSAP1) reversed the tumor-promoting effect of FOXP1 alone (Figures 5A–C). Furthermore, lactate content in tumor tissues was substantially higher in the oe-FOXP1+sh-NC group than in the control, and this increase was reduced in the oe-FOXP1+sh-NUSAP1 group compared with the FOXP1 overexpression alone group (Figure 5D).

IHC analysis showed upregulated protein expressions of FOXP1, NUSAP1, Ki67, and HK2 in the oe-FOXP1+sh-NC group. The upregulation of NUSAP1, Ki67, and HK2 was significantly inhibited upon NUSAP1 knockdown (Figure 5E). Additional results demonstrated that FOXP1 overexpression, by regulating NUSAP1, reshaped the immunosuppressive microenvironment, manifested as an increased proportion of M2 TAMs (Figure 5F) and elevated secretion of immunosuppressive factors Arg1 and IL-10 (Figures 5G,H). Moreover, TUNEL staining showed suppressed apoptosis of tumor cells overexpressing FOXP1 compared to control (Figure 5I). These phenomena were effectively reversed by NUSAP1 knockdown under FOXP1 overexpression conditions, indicating that targeting NUSAP1 can attenuate the immunosuppressive and anti-apoptotic capacities promoted by FOXP1 overexpression (Figures 5F–I). *In vivo* results demonstrate that FOXP1 accelerates HCC progression via the transcriptional activation of NUSAP1. This upregulation leads to increased glycolysis, TAM M2 polarization, enhanced tumor cell proliferation, and reduced apoptosis.

## 4 Discussion

This study reveals a novel mechanism by which the FOXP1/NUSAP1 axis promotes HCC progression by regulating glycolytic metabolic reprogramming in HCC cells to induce tumor-associated M2 TAM polarization. Experimental results demonstrate that TF FOXP1 activates NUSAP1 transcription, enhancing HCC cell glycolytic activity. Lactate produced via glycolysis induces TAM polarization towards the M2 phenotype, forming an immunosuppressive microenvironment that ultimately fosters HCC progression. This finding elucidates the critical role of the FOXP1/NUSAP1 axis in metabolic-immune crosstalk in HCC and simultaneously provides an important theoretical foundation for developing novel immunotherapeutic strategies targeting this signaling axis.

NUSAP1, a DNA replication regulation-related protein, promotes cell cycle transition, regulates stemness, controls DNA damage, and enhances proliferation and malignant progression in various cancers including HCC(Hu et al., 2022; Li et al., 2022; Chiu et al., 2023; Kong et al., 2023; Meng et al., 2024; Jiang et al., 2025). Furthermore, bioinformatics analysis suggested NUSAP1 expression correlates with CD4^+^ T cells and M0 TAM levels, potentially influencing these immune cells to promote HCC progression (Zhu et al., 2021). However, whether and how NUSAP1 affects HCC progression by regulating the TIME, particularly TAM functional phenotypes, requires experimental validation. Our study reveals NUSAP1 overexpression in HCC induces tumor-associated TAM M2 polarization, thereby promoting HCC cell malignant progression. This finding establishes NUSAP1 as a key connector between HCC cell-intrinsic oncogenic processes and the remodeling of the TIME.

To elucidate the mechanism by which NUSAP1 induces TAM M2 polarization in HCC, we confirmed that NUSAP1 promotes glycolytic metabolism in HCC via GSEA and cellular experiments. NUSAP1 has been confirmed to facilitate pancreatic cancer metastasis by activating LDHA-mediated glycolysis (Chen M. et al., 2023), indicating NUSAP1’s enormous potential to influence tumor progression by regulating glycolytic metabolic reprogramming. Notably, aerobic glycolysis in tumor cells is a key energy metabolic feature and plays a crucial role in shaping the immunosuppressive microenvironment (Reinfeld et al., 2022). This metabolic reprogramming leads to accumulation of metabolites such as lactate in the TME, and lactate has been shown to directly induce TAM polarization towards an immunosuppressive M2 phenotype through mechanisms such as activating the GPR132 receptor and stabilizing HIF-1α expression (Jin et al., 2025). Specifically, tumor-derived lactate can activate the GPR132 receptor on macrophages and trigger downstream cAMP–PKA signaling, thereby upregulating the transcription of M2-associated markers such as Arg1 and CD206 (Chen X. et al., 2025). In addition, accumulated lactate can stabilize HIF-1α under normoxic conditions in macrophages, thereby driving the expression of M2-polarization–related target genes (Colegio et al., 2014; Zhang and Li, 2020). Consistent with these mechanisms, our study demonstrates that NUSAP1 enhances glycolytic flux and lactate release from HCC cells, thereby driving TAM polarization toward the M2 phenotype and subsequently promoting HCC cell proliferation, migration, and invasion while inhibiting apoptosis. Notably, the glycolysis inhibitor 2-DG markedly reversed the NUSAP1-induced M2-polarization phenotype and secretion of M2-related cytokines, confirming that glycolysis served as an essential intermediate step through which NUSAP1 promoted TAM M2 polarization. These findings collectively highlight the critical role of NUSAP1-driven glycolysis in shaping the immunosuppressive microenvironment of HCC, suggesting that targeting the NUSAP1-glycolysis axis may be a potential strategy to improve HCC immunotherapy efficacy. However, the upstream regulatory mechanisms governing NUSAP1 expression warrant further investigation.

To dissect upstream regulatory mechanisms for NUSAP1 overexpression in HCC, bioinformatics analysis of potential upstream TFs identified FOXP1. FOXP1, a pluripotent TF, directly binds the NUSAP1 promoter region and significantly enhances its transcriptional activity, representing a key upstream regulator of NUSAP1 overexpression in HCC. Moreover, FOXP1 function exhibits tissue specificity. For example, low FOXP1 expression correlates with poor prognosis in pancreatic cancer patients, and its knockdown promotes lung cancer progression (Sheng et al., 2019; Wang et al., 2023). However, in HCC, silencing FOXP1 has been shown to markedly inhibit HCC cell growth both *in vitro* and *in vivo* (Wang et al., 2016). Furthermore, clinicopathological studies employing qRT-PCR, immunohistochemistry, and Kaplan–Meier survival analysis have further demonstrated that FOXP1 was significantly upregulated in HCC tissues compared with adjacent non-tumor tissues. Its high expression is closely associated with larger tumor diameter, elevated serum α-fetoprotein levels, and advanced tumor-node-metastasis stage, and has been identified as an independent prognostic factor, further supporting the oncogenic role of FOXP1 in HCC(Zhang et al., 2012). A systematic analysis based on the TCGA-LIHC cohort likewise indicates that most FOX family members, including FOXP1, are highly expressed in HCC tissues compared with normal liver tissue and are closely associated with tumor stage, grade, and overall survival (Ouyang et al., 2022). Together, these functional and clinical lines of evidence support the oncogenic role of FOXP1 in HCC. Recent research confirmed FOXP1 is significantly upregulated in TAMs in a Mir34a myeloid-specific knockout mouse model, accelerating colitis-associated colorectal cancer development (Konig et al., 2025). By upregulating FOXP1 and driving the transcription of GLUT4 and KHK, ac4C modification augments glycolytic flux in cervical cancer cells, thereby fueling tumor aggressiveness (Chen X. et al., 2023). These findings indicate FOXP1 may play important roles in regulating TAM function and tumor cell glycolytic metabolism. We demonstrated that FOXP1 significantly enhances NUSAP1 transcription in HCC by directly binding to its promoter. This upregulation triggers the glycolytic pathway, which subsequently drives TAM polarization toward the M2 phenotype and accelerates tumor progression, thereby underscoring the critical regulatory function of the FOXP1/NUSAP1 axis in HCC.

In conclusion, this study demonstrates a novel mechanism where FOXP1 transcriptionally activates NUSAP1 expression, promoting HCC cell glycolytic metabolic reprogramming, which in turn induces TAM M2 polarization. However, limitations exist. Firstly, the more detailed molecular mechanisms by which NUSAP1 regulates glycolysis remain to be elucidated; for instance, whether NUSAP1 directly binds key glycolytic enzymes such as LDHA, or modulates the expression of glycolytic genes through specific transcription factors, requires further investigation using approaches such as co-immunoprecipitation and integrated transcriptomic–metabolomic analyses. Secondly, whether NUSAP1 influences the function of other immune cell populations, such as T cells and NK cells, via glycolysis-related mechanisms warrants further exploration. Additionally, the clinical relevance of the FOXP1/NUSAP1/glycolysis axis needs further validation in HCC clinical tissue samples. Future research will focus on exploring the global regulatory role of glycolysis in the TIME and systematically analyzing the association of this metabolic signaling axis with patient prognosis and immunotherapy response through clinical sample collection.

## Data Availability

The original contributions presented in the study are included in the article/Supplementary Material, further inquiries can be directed to the corresponding author.

## References

[B1] Brar G. Greten T. F. Graubard B. I. McNeel T. S. Petrick J. L. McGlynn K. A. (2020). Hepatocellular carcinoma survival by etiology: a SEER-medicare database analysis. Hepatol. Commun. 4 (10), 1541–1551. 10.1002/hep4.1564 33024922 PMC7527688

[B2] Bray F. Laversanne M. Sung H. Ferlay J. Siegel R. L. Soerjomataram I. (2024). Global cancer statistics 2022: GLOBOCAN estimates of incidence and mortality worldwide for 36 cancers in 185 countries. CA Cancer J. Clin. 74 (3), 229–263. 10.3322/caac.21834 38572751

[B3] Caio G. Lungaro L. Caputo F. Zoli E. Giancola F. Chiarioni G. (2021). Nutritional treatment in crohn's disease. Nutrients 13 (5), 1628. 10.3390/nu13051628 34066229 PMC8151495

[B4] Chen M. Cen K. Song Y. Zhang X. Liou Y. C. Liu P. (2023a). NUSAP1-LDHA-Glycolysis-Lactate feedforward loop promotes warburg effect and metastasis in pancreatic ductal adenocarcinoma. Cancer Lett. 567, 216285. 10.1016/j.canlet.2023.216285 37354982

[B5] Chen X. Hao Y. Liu Y. Zhong S. You Y. Ao K. (2023b). NAT10/ac4C/FOXP1 promotes malignant progression and facilitates immunosuppression by reprogramming glycolytic metabolism in cervical cancer. Adv. Sci. (Weinh) 10 (32), e2302705. 10.1002/advs.202302705 37818745 PMC10646273

[B6] Chen X. Zhang Z. Wang K. (2025a). Lactate released by lung adenocarcinoma (LUAD) cells promotes M2 macrophage polarization *via* the GPR132/cAMP/PKA pathway. Genes Genomics 47 (5), 521–531. 10.1007/s13258-025-01622-1 40053234

[B7] Chen Y. Chen D. Liang Z. Yu H. Sun H. Hu Y. (2025b). Immune checkpoint inhibitors in hepatocellular carcinoma therapy: resistance mechanisms, liver transplantation challenges and management strategies. Cancer Drug Resist 8, 48. 10.20517/cdr.2025.120 41019983 PMC12462397

[B8] Chiu C. L. Li C. G. Verschueren E. Wen R. M. Zhang D. Gordon C. A. (2023). NUSAP1 binds ILF2 to modulate R-Loop accumulation and DNA damage in prostate cancer. Int. J. Mol. Sci. 24 (7), 6258. 10.3390/ijms24076258 37047232 PMC10093842

[B9] Colegio O. R. Chu N. Q. Szabo A. L. Chu T. Rhebergen A. M. Jairam V. (2014). Functional polarization of tumour-associated macrophages by tumour-derived lactic acid. Nature 513 (7519), 559–563. 10.1038/nature13490 25043024 PMC4301845

[B10] Feng J. Li J. Wu L. Yu Q. Ji J. Wu J. (2020). Emerging roles and the regulation of aerobic glycolysis in hepatocellular carcinoma. J. Exp. Clin. Cancer Res. 39 (1), 126. 10.1186/s13046-020-01629-4 32631382 PMC7336654

[B11] Hu Y. Xue Z. Qiu C. Feng Z. Qi Q. Wang J. (2022). Knockdown of NUSAP1 inhibits cell proliferation and invasion through downregulation of TOP2A in human glioblastoma. Cell Cycle 21 (17), 1842–1855. 10.1080/15384101.2022.2074199 35532155 PMC9359390

[B12] Ji W. Bai J. Ke Y. (2023). Exosomal ZFPM2-AS1 contributes to tumorigenesis, metastasis, stemness, macrophage polarization, and infiltration in hepatocellular carcinoma through PKM mediated glycolysis. Environ. Toxicol. 38 (6), 1332–1346. 10.1002/tox.23767 36880413

[B13] Jiang S. Li G. Peng S. Chen S. Pang Y. Cui H. (2025). PRMT1-catalyzed NUSAP1 methylation enhances Notch2 signaling and 5-FU resistance in gastric cancer. Cell Death Dis. 16 (1), 404. 10.1038/s41419-025-07723-9 40393971 PMC12092681

[B14] Jin X. Zhang N. Yan T. Wei J. Hao L. Sun C. (2025). Lactate-mediated metabolic reprogramming of tumor-associated macrophages: implications for tumor progression and therapeutic potential. Front. Immunol. 16, 1573039. 10.3389/fimmu.2025.1573039 40433363 PMC12106438

[B15] Kong J. Xu S. Zhang P. Wang Y. (2023). Transcription factor E2F8 promotes cisplatin resistance in hepatocellular carcinoma by regulating DNA damage *via* NUSAP1. Int. J. Toxicol. 42 (5), 420–429. 10.1177/10915818231182114 37331996

[B16] Konig J. Rokavec M. Oner-Ziegler M. G. Fei Y. Hermeking H. (2025). Myeloid Mir34a suppresses colitis-associated Colon cancer: characterization of mediators by single-cell RNA sequencing. Cell Death Differ. 32 (2), 225–241. 10.1038/s41418-024-01380-9 39425000 PMC11802797

[B17] Ladd A. D. Duarte S. Sahin I. Zarrinpar A. (2024). Mechanisms of drug resistance in HCC. Hepatology 79 (4), 926–940. 10.1097/HEP.0000000000000237 36680397

[B18] Li J. Tang M. Wu J. Qu H. Tu M. Pan Z. (2022). NUSAP1, a novel stemness-related protein, promotes early recurrence of hepatocellular carcinoma. Cancer Sci. 113 (12), 4165–4180. 10.1111/cas.15585 36106345 PMC9746044

[B19] Meng J. Yang Z. Jiang X. Zou J. (2024). Unveiling NUSAP1 as a common gene signature linking chronic HBV infection and HBV-Related HCC. Discov. Oncol. 15 (1), 61. 10.1007/s12672-024-00922-4 38441732 PMC10914659

[B20] Ouyang X. Feng L. Yao L. Zhang J. Xiao Y. Liu G. (2022). A comprehensive analysis of FOX family in HCC and experimental evidence to support the oncogenic role of FOXH1. Aging (Albany NY) 14 (5), 2268–2286. 10.18632/aging.203934 35255005 PMC8954963

[B21] Reinfeld B. I. Rathmell W. K. Kim T. K. Rathmell J. C. (2022). The therapeutic implications of immunosuppressive tumor aerobic glycolysis. Cell Mol. Immunol. 19 (1), 46–58. 10.1038/s41423-021-00727-3 34239083 PMC8752729

[B22] Seyedian S. S. Nokhostin F. Malamir M. D. (2019). A review of the diagnosis, prevention, and treatment methods of inflammatory bowel disease. J. Med. Life 12 (2), 113–122. 10.25122/jml-2018-0075 31406511 PMC6685307

[B23] Sheng H. Li X. Xu Y. (2019). Knockdown of FOXP1 promotes the development of lung adenocarcinoma. Cancer Biol. Ther. 20 (4), 537–545. 10.1080/15384047.2018.1537999 30409062 PMC6422458

[B24] Strizova Z. Benesova I. Bartolini R. Novysedlak R. Cecrdlova E. Foley L. K. (2023). M1/M2 macrophages and their overlaps - myth or reality? Clin. Sci. (Lond) 137 (15), 1067–1093. 10.1042/CS20220531 37530555 PMC10407193

[B25] Tian Z. Hou X. Liu W. Han Z. Wei L. (2019). Macrophages and hepatocellular carcinoma. Cell Biosci. 9, 79. 10.1186/s13578-019-0342-7 31572568 PMC6761725

[B26] Veauthier B. Hornecker J. R. (2018). Crohn's disease: diagnosis and management. Am. Fam. Physician 98 (11), 661–669. 30485038

[B27] Wang X. Sun J. Cui M. Zhao F. Ge C. Chen T. (2016). Downregulation of FOXP1 inhibits cell proliferation in hepatocellular carcinoma by inducing G1/S phase cell cycle arrest. Int. J. Mol. Sci. 17 (9), 1501. 10.3390/ijms17091501 27618020 PMC5037778

[B28] Wang L. Luo P. Yang Z. Zhong X. Ji C. (2023). FOXP1 inhibits pancreatic cancer growth by transcriptionally regulating IRF1 expression. PLoS One 18 (3), e0280794. 10.1371/journal.pone.0280794 36952469 PMC10035899

[B29] Xu B. Liu Y. Li N. Geng Q. (2024). Lactate and lactylation in macrophage metabolic reprogramming: current progress and outstanding issues. Front. Immunol. 15, 1395786. 10.3389/fimmu.2024.1395786 38835758 PMC11148263

[B30] Xu S. Nan D. Liu R. Liu C. Hu Y. Huang L. (2025). The role and therapeutic value of NUSAP1 in human cancers. J. Transl. Med. 23 (1), 725. 10.1186/s12967-025-06746-2 40605080 PMC12220482

[B31] Yang J. D. Hainaut P. Gores G. J. Amadou A. Plymoth A. Roberts L. R. (2019). A global view of hepatocellular carcinoma: trends, risk, prevention and management. Nat. Rev. Gastroenterol. Hepatol. 16 (10), 589–604. 10.1038/s41575-019-0186-y 31439937 PMC6813818

[B32] Yang C. Zhang H. Zhang L. Zhu A. X. Bernards R. Qin W. (2023). Evolving therapeutic landscape of advanced hepatocellular carcinoma. Nat. Rev. Gastroenterol. Hepatol. 20 (4), 203–222. 10.1038/s41575-022-00704-9 36369487

[B33] Yang Y. Li S. To K. K. W. Zhu S. Wang F. Fu L. (2025). Tumor-associated macrophages remodel the suppressive tumor immune microenvironment and targeted therapy for immunotherapy. J. Exp. Clin. Cancer Res. 44 (1), 145. 10.1186/s13046-025-03377-9 40380196 PMC12083052

[B34] Zeng T. Chen G. Qiao X. Chen H. Sun L. Ma Q. (2022). NUSAP1 could be a potential target for preventing NAFLD progression to liver cancer. Front. Pharmacol. 13, 823140. 10.3389/fphar.2022.823140 35431924 PMC9010788

[B35] Zhang L. Li S. (2020). Lactic acid promotes macrophage polarization through MCT-HIF1alpha signaling in gastric cancer. Exp. Cell Res. 388 (2), 111846. 10.1016/j.yexcr.2020.111846 31945319

[B36] Zhang Y. Zhang S. Wang X. Liu J. Yang L. He S. (2012). Prognostic significance of FOXP1 as an oncogene in hepatocellular carcinoma. J. Clin. Pathol. 65 (6), 528–533. 10.1136/jclinpath-2011-200547 22422806

[B37] Zhang Q. Liu W. Zhang H. M. Xie G. Y. Miao Y. R. Xia M. (2020). hTFtarget: a comprehensive database for regulations of human transcription factors and their targets. Genomics Proteomics Bioinforma. 18 (2), 120–128. 10.1016/j.gpb.2019.09.006 32858223 PMC7647694

[B38] Zhang S. Zhang X. Huang W. Jiang G. Mo Y. Wei L. (2024). NUSAP1 is upregulated by estrogen to promote lung adenocarcinoma growth and serves as a therapeutic target. Int. J. Biol. Sci. 20 (13), 5375–5395. 10.7150/ijbs.100188 39430250 PMC11489181

[B39] Zheng H. Wang M. Zhang S. Hu D. Yang Q. Chen M. (2023). Comprehensive pan-cancer analysis reveals NUSAP1 is a novel predictive biomarker for prognosis and immunotherapy response. Int. J. Biol. Sci. 19 (14), 4689–4708. 10.7150/ijbs.80017 37781040 PMC10535697

[B40] Zhu W. Xu J. Chen Z. Jiang J. (2021). Analyzing roles of NUSAP1 from clinical, molecular mechanism and immune perspectives in hepatocellular carcinoma. Front. Genet. 12, 689159. 10.3389/fgene.2021.689159 34354737 PMC8329558

